# The Glucagon-Like Peptide 1 Analogue, Exendin-4, Attenuates the Rewarding Properties of Psychostimulant Drugs in Mice

**DOI:** 10.1371/journal.pone.0069010

**Published:** 2013-07-16

**Authors:** Emil Egecioglu, Jörgen A. Engel, Elisabet Jerlhag

**Affiliations:** Institute of Neuroscience and Physiology, Department of Pharmacology, the Sahlgrenska Academy at the University of Gothenburg, Gothenburg, Sweden; University of Ulster, United Kingdom

## Abstract

Glucagon-like peptide 1 (GLP-1) is an incretine hormone that controls consummatory behavior and glucose homeostasis. It is released in response to nutrient ingestion from the intestine and production in the brain has also been identified. Given that GLP-1 receptors are expressed in reward areas, such as the nucleus accumbens and ventral tegmental area, and that common mechanisms regulate food and drug-induced reward we hypothesize that GLP-1 receptors are involved in reward regulation. Herein the effect of the GLP-1 receptor agonist Exendin-4 (Ex4), on amphetamine- and cocaine-induced activation of the mesolimbic dopamine system was investigated in mice. In a series of experiments we show that treatment with Ex4, at a dose with no effect *per se*, reduce amphetamine- as well as cocaine-induced locomotor stimulation, accumbal dopamine release as well as conditioned place preference in mice. Collectively these data propose a role for GLP-1 receptors in regulating drug reward. Moreover, the GLP-1 signaling system may be involved in the development of drug dependence since the rewarding effects of addictive drugs involves interferences with the mesolimbic dopamine system. Given that GLP-1 analogues, such as exenatide and liraglutide, are clinically available for treatment of type II diabetes, we propose that these should be elucidated as treatments of drug dependence.

## Introduction

Glucagon-like peptide 1 (GLP-1), an incretine gut-brain hormone, is released in response to nutrient ingestion [1] from enteroendocrine L-cells of the intestinal mucosa [2]. In addition, GLP-1 containing neurons in the nucleus tractus solitarius (NTS) project throughout the brain [3–6] and for instance target areas involved in energy balance homeostasis such as the hypothalamus [7]. In accordance, GLP-1 was initially shown to regulate food intake and consummatory behavior in rodents [8–10]. In addition to energy balance regulation GLP-1 receptors control glucose-dependent insulin secretion [11], gastric emptying and glucagon secretion [12–14]. Hence, GLP-1 analogues have been approved and are today used in the treatment of type II diabetes. Given that GLP-1 neurons in the NTS project to reward related areas expressing GLP-1 receptors such as the ventral tegmental area and nucleus accumbens [3,4], a novel role for GLP-1 in reward regulation may be suggested.

Usage of psychostimulants, such as amphetamine and cocaine, may cause addiction and the addiction related costs are significant for both society and the individuals [15,16]. Drug addiction, a chronic relapsing disorder, is characterized by loss of control, compulsive drug-seeking and intake despite adverse consequences [17]. Its development largely depends on that the rewarding effects of additive drugs interfere with the mesolimbic dopamine system (for review, see 18–20). By elucidating the complex neurochemical mechanisms involved in the ability of addictive drugs to activate the mesolimbic dopamine system novel treatment strategies for drug dependence can be developed. The present series of experiments was designed to evaluate the effects of a GLP-1 analogue, exendin-4 (Ex4), on the ability of amphetamine and cocaine to activate the mesolimbic dopamine system as measured by locomotor stimulation, accumbal dopamine release and conditioned place preference (CPP) in mice.

## Material and Methods

### Animals

Adult post-pubertal age-matched male NMRI mice (8-12 weeks old and 25-40 g body weight; Charles River, Sulzfeld, Germany) were used as described previously [21]. In brief, all mice were group housed and maintained at a 12/12 hour light/dark cycle (lights on at seven am). Tap water and food (Normal chow; Harlan Teklad, Norfolk, England) were supplied *ad libitum*, except during the experimental setups. The study was carried out in strict accordance with the recommendations in the Swedish Animal Welfare Act and all experiments were approved by the Swedish Ethical Committee on Animal Research in Gothenburg (permit numbers: 81-07, 80-07, 117-11 and 26-12). All efforts were made to minimize animal suffering, and to reduce the number of animals used. All animals were allowed to acclimatize at least one week before the start of the experiments.

### Drugs

Dex-amphetamine sulphate (RBI, Natick, USA) was dissolved in vehicle (0.9% sodium chloride solution) and was administered i.p. at a dose of 2 mg/kg 10 minutes prior to initiation of the experiment. Cocaine (Sigma; St Louise, USA) was dissolved in the vehicle (0.9% sodium chloride solution) and was administered i.p. at a dose of 10 mg/kg 10 minutes prior to initiation of the experiment. Similar doses have been used previously to induce an activation of the mesolimbic dopamine system as measured by locomotor activity and accumbal dopamine release in rats [22]. The peptide Ex4 (Tocris Bioscience, Bristol, UK) has previously been established as a GLP-1 receptor agonist [23] and distribution studies show that the CNS binding of Ex4 is identical to GLP-1 [24]. The Ex4 dose (2.4 µg/kg) was since this dose previously was shown to be the highest dose that did not affect locomotor activity *per se* as well as blocked the rewarding properties of alcohol in mice [25]. In all experiments the selected dose of Ex4 did not affect the mice gross behavior. Exendin-4 was dissolved in vehicle (0.9% sodium chloride) and stored in aliquots at -20 degrees C before use. The Ex4 stock-solution was thawed before each treatment occasion and administered intra peritoneal (IP) 10 minutes prior the start of each experiment. A balanced or within subject design was used for all drug challenges.

### Locomotor activity experiments

Locomotor activity was recorded as described previously [26]. In brief, locomotor activity was registered in eight sound attenuated, ventilated and dim lit locomotor boxes (420 x 420 x 200 mm, Kungsbacka mät- och reglerteknik AB, Fjärås, Sweden). Five by five rows of photocell beams, at the floor level of the box, creating photocell detection allowed a computer-based system to register the activity of the mice. Locomotor activity was defined as the accumulated number of new photocell beams interrupted during a 60 minute period.

The mice were allowed to habituate to the locomotor activity box one hour prior to drug challenge. In the first series of experiment the effects of Ex4 (2.4 µg/kg, IP) on amphetamine-induced (2 mg/kg, IP) locomotor stimulation were investigated. Ex4 was administered ten minutes prior to amphetamine and the activity registration started ten minutes after the last injection. Each mouse received one treatment combination (vehicle/vehicle, Ex4/vehicle, amphetamine/vehicle or Ex4/amphetamine; n=8 per treatment combination) and was only subjected to one experimental trial. In a subsequent series of experiment, in separate mice, the effects of Ex4 (2.4 µg/kg, IP) on cocaine-induced (10 mg/kg, IP) locomotor stimulation were investigated. Ex4 was administered ten minutes prior to cocaine and the activity registration started ten minutes after the last injection. Each mouse received one treatment combination (vehicle/vehicle, Ex4/vehicle, cocaine/vehicle or Ex4/cocaine; n=8 per treatment combination) and was only subjected to one experimental trial.

### In vivo microdialysis and dopamine release measurements

For measurements of extracellular dopamine levels, mice were implanted unilaterally with a microdialysis probe positioned in the nucleus accumbens. The surgery was performed as described in detail previously [26]. In brief, the mice were anesthetized with isofluran (Isofluran Baxter; Univentor 400 Anaesthesia Unit, Univentor Ldt., Zejtun, Malta), placed in a stereotaxic frame (David Kopf Instruments, Tujunga, CA, USA) and kept on a heating pad to prevent hypothermia. The skull bone was exposed and one hole for the probe and one for the anchoring screw were drilled. The probe was randomly alternated to either the left or right side of the brain. The coordinates of 1.5 mm anterior to the bregma, ±0.7 lateral to the midline and 4.7 mm below the surface of the brain surface was used for the nucleus accumbens [27]. The exposed tip of the dialysis membrane (20 000 kDa cut off with an o.d./i.d. of 310/220 µm, HOSPAL, Gambro, Lund, Sweden) of the probe was 1 mm. All probes were surgically implanted two days prior to the experiment. After surgery the mice were kept in individual cages (Macrolon III).

In the first series of experiments the effect of systemic administration of Ex4 (2.4 µg/kg, IP) on amphetamine-induced (2 mg/kg, IP) accumbal dopamine release was investigated using microdialysis in freely moving mice. In subsequent experiments, in separate mice, the effects of Ex4 (2.4 µg/kg, IP) on cocaine-induced (10 mg/kg, IP) accumbal dopamine release was investigated. As described below a similar procedure for both these series of experiments was used.

On the test day the probe was connected to a microperfusion pump (U-864 Syringe Pump; AgnThós AB) and perfused with Ringer solution at a rate of 1.5 µl/minute. After one hour of habituation to the microdialysis set-up, perfusion samples were collected every 20 minutes. The baseline dopamine level was defined as the average of three consecutive samples before the first drug/vehicle challenge, and the increase in accumbal dopamine was calculated as the percent increase from baseline. After the baseline samples (-40 minutes until 0 minutes), mice were injected with Ex4 or vehicle (at 10 minutes), which was followed by an amphetamine or vehicle injection (at 20 minutes). In the second series of experiments mice were initially injected with Ex4 or vehicle (at 10 minutes), which was followed by a second injection containing either cocaine or vehicle (at 20 minutes). Following these drug administrations an additional eight 20 minute samples were collected. Collectively the following treatment groups (n=8 in each group) were created. In the first series of experiments: vehicle-vehicle (Veh-Veh), vehicle-amphetamine (Veh-Amph), Ex4-vehicle (Ex4-Veh) and Ex4-amphetamine (Ex4-Amph). In the second series of experiments: vehicle-vehicle (Veh-Veh), vehicle-cocaine (Veh-Coc), Ex4-vehicle (Ex4-Veh) and Ex4-cocaine (Ex4-Coc).

The dopamine levels in the dialysates were determined by HPLC with electrochemical detection. A pump (Gyncotec P580A; Kovalent AB; V. Frölunda, Sweden), an ion exchange column (2.0 x 100 mm, Prodigy 3 µm SA; Skandinaviska GeneTec AB; Kungsbacka, Sweden) and a detector (Antec Decade; Antec Leyden; Zoeterwoude, The Netherlands) equipped with a VT-03 flow cell (Antec Leyden) were used. The mobile phase (pH 5.6), consisting of sulfonic acid 10 mM, citric acid 200 mM, sodium citrate 200 mM, 10% EDTA, 30% MeOH, was vacuum filtered using a 0.2 µm membrane filter (GH Polypro; PALL Gelman Laboratory, Lund, Sweden). The mobile phase was delivered at a flow rate of 0.2 ml/minute passing a degasser (Kovalent AB), and the analyte was oxidized at +0.4 V.

After the microdialysis experiments were completed, the mice were decapitated, and probes were perfused with pontamine sky blue 6BX to facilitate probe localization. The brains were mounted on a vibroslice device (752M Vibroslice; Campden Instruments Ltd., Loughborough, UK) and cut in 50 µm sections. The location of the probe was determined by gross observation using light microscopy. The exact position of the probe was verified [27] and only mice with correct placements were used in the statistical analysis.

### Conditioned place preference

To evaluate the effects of Ex4 on the rewarding effects of either amphetamine or cocaine, CPP tests were performed in mice as previously described [28]. In brief, a two-chambered CPP apparatus, with 45 lux illumination and distinct visual and tactile cues was used. One compartment was defined by black and white striped walls and by a dark laminated floor whereas the other had a white painted wooden floor and walls of wooden texture. The procedure consisted of pre-conditioning (day 1), conditioning (days 2-5), and post-conditioning (day 6). At preconditioning, mice were injected IP with vehicle and was placed in the chamber with free access to both compartments during 20 minutes to determine the initial place (or side) preference. Conditioning (20 minutes per session) was done using a biased procedure in which amphetamine (2 mg/kg) was paired with the least preferred compartment and vehicle with the preferred compartment. All mice received one amphetamine and one vehicle injection every day and the injections were altered between morning and afternoon in a balances design. At post-conditioning, mice were injected with Ex4 (2.4 µg/kg, IP) or an equal volume of vehicle solution and 10 minutes later placed on the midline between the two compartments with free access to both compartments for 20 minutes (creating the following treatment groups; Amph-Veh and Amph-Ex4). The second series of experiments, conducted in separate mice, were conducted as above however cocaine was used instead of amphetamine (creating the following treatment groups; Coc-Veh and Coc-Ex4). Previous control experiments have shown that the selected dose of Ex4 does not induce a CPP *per se* [25].

Condition place preference was calculated as the difference in % of total time spent in the drug-paired (i.e. least preferred) compartment during the post-conditioning and the pre-conditioning session.

### Statistical analysis

Locomotor activity data was evaluated by a one-way ANOVA followed by Bonferroni post-hoc tests. The microdialysis experiments were evaluated by a two-way ANOVA followed by Bonferroni post-hoc test for comparisons between different treatments and specifically at given time points. The condition place preference data were evaluated by an unpaired t-test. Data are presented as mean ± SEM. A probability value of P<0.05 was considered as statistically significant.

## Results

### Effects of Ex4 on amphetamine-induced locomotor stimulation, accumbal dopamine release and conditioned place preference in mice

**Figure 1:**
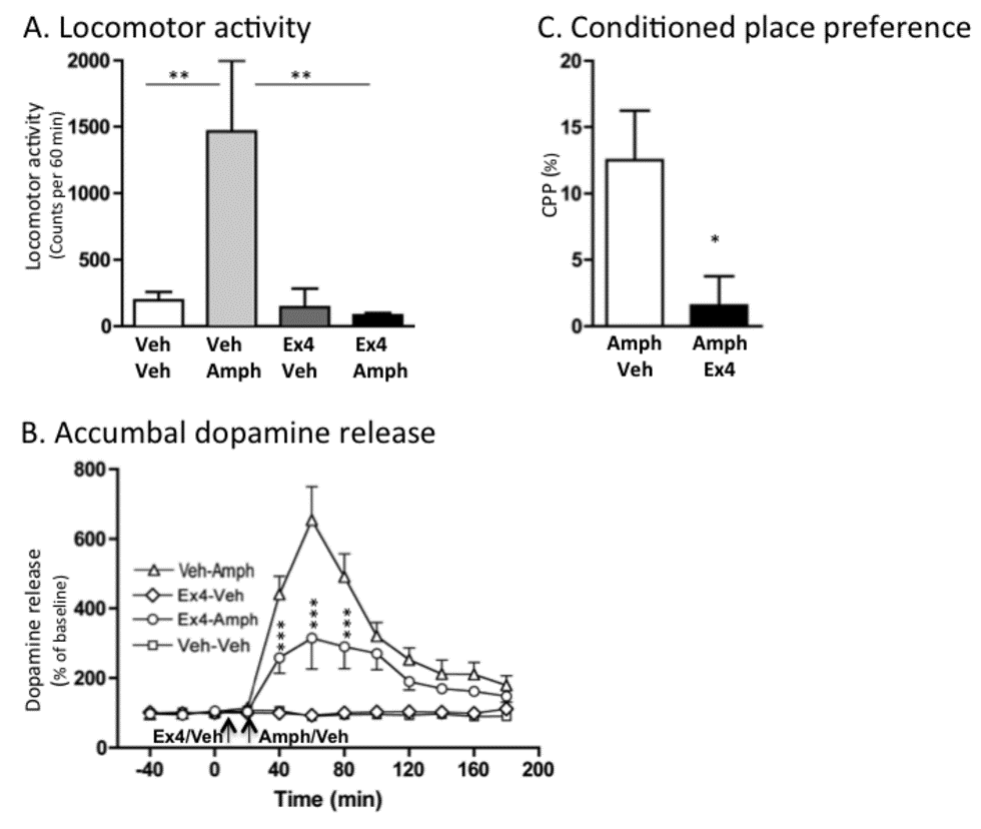
Exendin-4 attenuates amphetamine-induced locomotor stimulation, accumbal dopamine release and conditioned place preference in mice. (A) Amphetamine-induced (2 mg/kg IP) locomotor stimulation was attenuated by a single injection of Ex4 (2.4 µg/kg IP) (n=8 in each group; **P<0.01, one-way ANOVA followed by a Bonferroni post-hoc test). (B) First we demonstrated a significant effect of amphetamine (2 mg/kg IP) to increase dopamine release in comparison to vehicle treatment (time interval 40-120 minutes (P<0.001) and 140-160 minutes (P<0.05), Veh-Veh vs Veh-Amph). As shown in (B) pre-treatment with Ex4 (2.4 µg/kg IP) attenuated the amphetamine-induced increase in dopamine release compared to vehicle pre-treatment at time interval 40-8 minutes (*** < 0.001, Ex4-Amph compared to Veh-Amph treatment). The selected dose had no significant effect on accumbal dopamine release compared to vehicle treatment at any time interval (*P*>0.05, Veh-Veh vs Ex4-Veh). There was a significant difference in accumbal dopamine response in vehicle treated mice and Ex4-Amph treated mice at time interval 40-100 minutes (*P*>0.001). Arrows represent time points of injection of Ex4, vehicle and amphetamine. Data analyzed with a Two-way ANOVA followed by a Bonferroni post-hoc test (n=8 in each group) (C) The amphetamine-induced (2 mg/kg IP) condition place preference (CPP) was attenuated by an acute single IP injection of Ex4 (2.4 µg/kg IP) in mice (n=8 in each group, *P<0.05, unpaired t-test). All values represent mean ± SEM.

An overall main effect of treatment was found on locomotor activity in mice following systemic administration of amphetamine (2 mg/kg) and Ex4 (2.4 µg/kg) (F(3,28)=6.03, *P*=0.0027; n=8 per group). As shown in Figure 1A, post hoc analysis revealed that amphetamine significantly increased the locomotor activity compared to vehicle (*P*<0.01). This amphetamine-induced locomotor activity was significantly reduced by pre-treatment with a single injection of Ex4 (P<0.01), at a dose that alone had no significant effect on locomotor activity compared to vehicle treatment (*P*>0.05). There was no difference in locomotor activity response in vehicle treated mice and Ex4-Amph treated mice (*P*>0.05).

Accumbal microdialysis measurements of dopamine in mice revealed an overall main effect of treatment (F(3,33)=206.50, P<0.0001), F(11,252)=12.00, P<0.0001) and treatment x time interaction (F(11,252)=10.65, P<0.0001) (Figure 1B; n=8 in each group). Amphetamine increased accumbal dopamine release relative to vehicle treatment at time interval 40-120 minutes (P<0.001) and 140-160 minutes (P<0.05). As shown in Figure 1B this effect was reduced by pre-treatment with Ex4 at time interval 40-80 minutes (P<0.001). The selected dose had no significant effect on accumbal dopamine release compared to vehicle treatment at any time interval (*P*>0.05). There was a significant difference in accumbal dopamine response in vehicle treated mice and Ex4-Amph treated mice at time interval 40-100 minutes (*P*>0.001).

The amphetamine-induced (2 mg/kg) (Amph-Veh) CPP was significantly attenuated by an acute single injection of Ex4 (2.4 µg/kg) (Amph-Ex4) on the post-conditioning day compared to vehicle injection (*P*=0.025, n=8 in each group; Figure 1C).

### Effects of Ex4 on cocaine-induced locomotor stimulation, accumbal dopamine release and conditioned place preference in mice

An overall main effect of treatment was found on locomotor activity in mice following systemic administration of cocaine (10 mg/kg) and Ex4 (2.4 µg/kg) (F(3,27)=9.63, *P*=0.0002; n=8 per group). As shown in Figure 1A, post hoc analysis revealed that cocaine significantly increased the locomotor activity compared to vehicle (*P*<0.001). This cocaine-induced locomotor activity was significantly reduced by pre-treatment with a single injection of Ex4 (P<0.05), at a dose that alone had no significant effect on locomotor activity compared to vehicle treatment (*P*>0.05). There was no difference in locomotor activity response in vehicle treated mice and Ex4-Coc treated mice (*P*>0.05).

**Figure 2:**
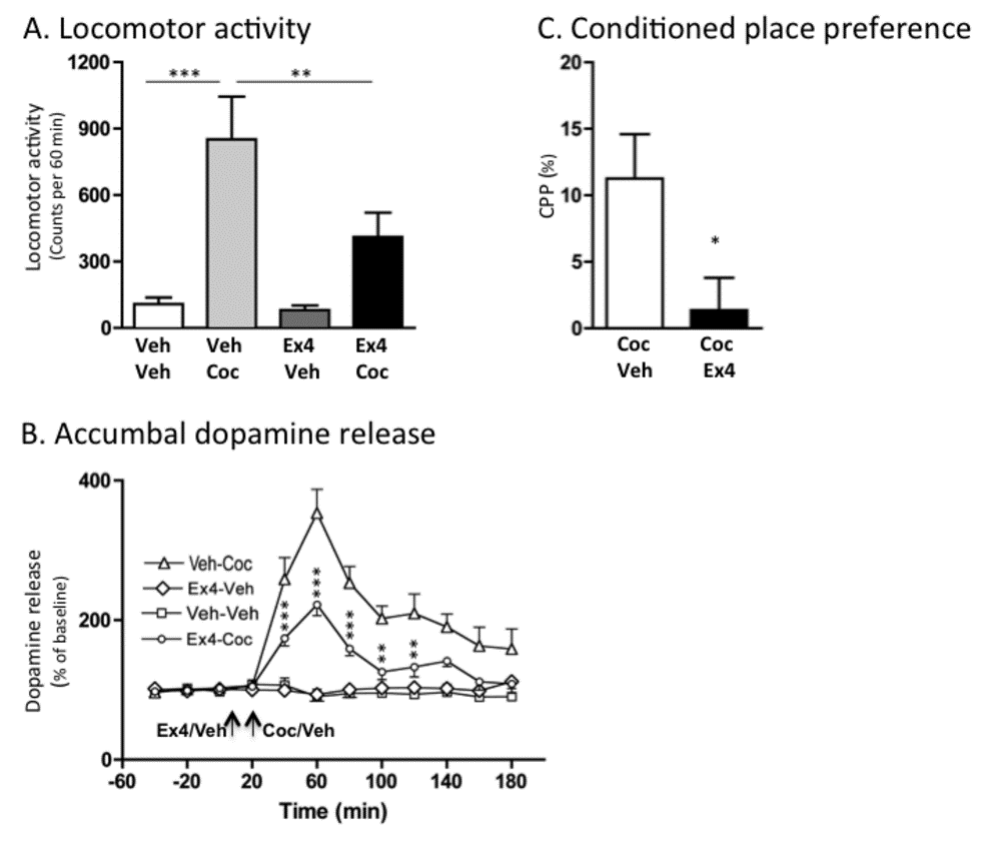
Exendin-4 attenuates cocaine-induced locomotor stimulation, accumbal dopamine release and conditioned place preference in mice. (A) Cocaine-induced (10 mg/kg IP) locomotor stimulation was attenuated by a single injection of Ex4 (2.4 µg/kg IP) (n=8 in each group; **P<0.01 and **P<0.001, one-way ANOVA followed by a Bonferroni post-hoc test). (B) First we demonstrated a significant effect of cocaine (10 mg/kg IP) to increase dopamine release in comparison to vehicle treatment (40-140 minutes (P<0.001) and 160-180 minutes (P<0.01) Veh-Veh vs Veh-Coc). As shown in (B) pre-treatment with Ex4 (2.4 µg/kg IP) attenuated the cocaine-induced increase in dopamine release compared to vehicle pre-treatment (** < 0.001 and *** < 0.001, Ex4-Coc compared to Veh-Coc treatment). The selected dose had no significant effect on accumbal dopamine release compared to vehicle treatment at any time interval (*P*>0.05, Veh-Veh vs Ex4-Veh). There was a significant difference in accumbal dopamine response in vehicle treated mice and Ex4-Coc treated mice (time interval 40 minutes (P<0.05), 60 minutes (P<0.001) and 80 minutes (P<0.05)). Arrows represent time points of injection of Ex4, vehicle and cocaine. Data analyzed with a Two-way ANOVA followed by a Bonferroni post-hoc test (n=8 in each group) (C) The cocaine-induced (10 mg/kg IP) condition place preference (CPP) was attenuated by an acute single IP injection of Ex4 (2.4 µg/kg IP) in mice (n=8 in each group, *P<0.05, unpaired t-test). All values represent mean ± SEM.

Accumbal microdialysis measurements of dopamine in mice revealed an overall main effect of treatment (F(3,33)=95.00, P<0.0001), time F(11,252)=29.35, P<0.0001) and treatment x time interaction (F(11,252)=7.663, P<0.0001) (Figure 1B; n=8 in each group). Cocaine increased accumbal dopamine release relative to vehicle treatment at time interval 40-140 minutes (P<0.001) and 160-180 minutes (P<0.01). As shown in Figure 2B this effect was attenuated by pre-treatment with Ex4 at time interval 40-80 minutes (P<0.001) and 100-120 minutes (P<0.01). The selected dose had no significant effect on accumbal dopamine release compared to vehicle treatment at any time interval (*P*>0.05). There was a significant difference in accumbal dopamine response in vehicle treated mice and Ex4-Coc treated mice at time interval 40 minutes (P<0.05), 60 minutes (P<0.001) and 80 minutes (P<0.05).

The cocaine-induced (10 mg/kg) (Coc-Veh) CPP was significantly attenuated by an acute single injection of Ex4 (2.4 µg/kg) (Coc-Ex4) on the post-conditioning day compared to vehicle injection (*P*=0.031, n=8 in each group; Figure 2C).

## Discussion

The present study demonstrates that the physiological role of GLP-1 receptors extends beyond control of food intake and glucose homeostasis and includes reward regulation. Hence, we show for the first time that the ability of amphetamine and cocaine to induce a locomotor stimulation, accumbal dopamine release and to condition a place preference is reduced in mice treated peripherally with the GLP-1 analogue, Ex4.

Albeit Ex4 was administered peripherally in the present study we hypothesize that the ability of Ex4 to attenuate the rewarding properties of psychostimulant drugs are mediated via GLP-1 receptors expressed within the mesolimbic dopamine system. Indeed, Ex4 readily passes the blood brain barrier [29]. Moreover, GLP-1 containing neurons from the NTS target reward related areas expressing GLP-1 receptors, such as the ventral tegmental area and nucleus accumbens [3,4,30,31]. It has also been found that Ex4 have effects within these reward areas since local administration in either area reduces the intake of as well as the reward induced by palatable foods [31,32]. In accordance, accumbal administration of a GLP-1 receptor antagonist causes hyperphagia [33]. The possibility that GLP-1 receptors within these reward areas interfere with the ability of psychostimulant drugs to increase the release or synthesis of accumbal dopamine or enhance the activity of mesolimbic dopamine neurons should be considered [34,35]. Another possibility is that Ex4 could cause alterations of the dopamine transporter or receptor function and thereby attenuates the rewarding properties of psychostimulant drugs [36,37]. However, downstream mechanisms or independent of the mesolimbic dopamine system may also be involved. Indeed, GLP-1 receptors found in the hypothalamus and NTS regulate GLP-1 dependent food intake [7,38] as well as glucose homeostasis [39]. In addition, peripheral GLP-1 receptors, for instance expressed on vagal neurons, are important for GLP-1 controlled food intake [40]. In conclusion, the exact mechanisms through which Ex4 reduce psychostimulant induced reward needs to be elucidated.

Given that Ex4, at a dose that has no effect per se, reduces the amphetamine- as well as cocaine-induced reward we suggest that GLP-1 receptors have an important role in reward regulation. However, a similar dose of Ex4 caused a condition taste aversion [41], raising the possibility that our results are due to aversion [42]. However, this appears less likely since in contrast to lithium chloride a higher dose of Ex4 does not cause behavioral aversion [43] and since repeated Ex4 treatment does not induce a conditioned place aversion in mice [25]. In addition, the present study show that Ex4 attenuates parameters reflecting reward rather than aversion, namely locomotor stimulation, CPP as well as accumbal dopamine release. Collectively, this suggests that activation of GLP-1 receptors in our experimental setups does not induce aversion and that the observed reduction in psychostimulant reward occurs in the absence of such a process. Given that Ex-4 reduces reward induced by alcohol as well as addictive drugs one could expect that GLP-1 analogues such as exenatide and lirglutide, which are approved for diabetes mellitus, should have anhedonic properties. However, clinical findings show that these dugs do not appear to have such effects. Additional studies, with higher doses of Ex4, are warranted to study the possible anhedonic properties of GLP-1 analogues. In the present study a dose of Ex4 with no effect *per se* on accumbal dopamine release, conditioned place preference and locomotor activity was used, and the selected dose should therefore not be anhedonic.

The ability of addictive drugs to cause stimulation, dopamine release and to induce a CPP are intimately associated with the reinforcing properties of addictive drugs and these parameters are considered to constitute a part of the addiction process [22,44–46]. Given that we here found that these effects of amphetamine and cocaine were attenuated by a dose of Ex4 that had no effect *per se*, we hypothesize that GLP-1 receptors may play an important role in addiction processes. Supportively, Ex4, at a significantly higher dose, blocks the ability of amphetamine to cause a locomotor stimulation and cocaine to induce a CPP in rodents [43,47]. Moreover, a low dose of Ex4 suppresses alcohol-induced reward as well as reduces alcohol consumption and alcohol seeking behavior [25]. The findings that activation of accumbal GLP-1 receptors reduces the intake of palatable food [33] as well as the motivation to consume sucrose in rodents [32], imply that GLP-1 receptors are important for the hedonic aspects of food intake. We therefore suggest that GLP-1 receptors have an important role in reward regulation in general. Collectively these data support the hypothesize that common mechanisms, including gut-brain hormones, control food intake as well as the responses to drugs of abuse [48]. Indeed, the orexigenic peptide ghrelin increases the behavioral responses to addictive drugs [49,50] and antagonism of its receptor reduces drug reward [51,52]. Moreover, the anorectic peptides orexin and leptin blocks reward induced by psychostimulant drugs [53,54].

Our present findings show for the first time that Ex4, at a dose with no effect *per se*, attenuates the psychostimulant-induced reward as measured by locomotor stimulation, accumbal dopamine release and CPP. Given that GLP-1 analogues, such as exenatide and liraglutide, are clinically available for treatment of type II diabetes, we propose that these should be elucidated as treatment of drug dependence.
